# Surface Treatment and Analysis of 3D-Printed Plastic Molds for Prototype and Small-Series Injection Molding

**DOI:** 10.3390/polym17222977

**Published:** 2025-11-08

**Authors:** Karel Raz, Zdenek Chval, Frantisek Hula, Angelos Markopoulos

**Affiliations:** 1 Faculty of Mechanical Engineering, Regional Technological Institute, University of West Bohemia, Univerzitni 2732/8, 301 00 Plzen, Czech Republic; zdchval@fst.zcu.cz; 2 3Dees, Doudlebska 1699/5, 140 00 Praha, Czech Republic; frantisek.hula@3dees.cz; 3 School of Mechanical Engineering, National Technical University of Athens, Heroon Polytechniou 9 Zografou Campus, 15780 Athens, Greece; amark@mail.ntua.gr

**Keywords:** additive manufacturing, 3D printing, injection molding, surface treatment, roughness analysis, tribology, prototype tooling

## Abstract

Additive manufacturing (AM) has emerged as a promising technology for producing low-cost, customized tooling, particularly for prototyping and small-series injection molding. However, the inherent surface roughness and anisotropic properties of 3D-printed parts pose significant challenges for their direct use as functional mold inserts. This study investigates the effectiveness of various post-processing techniques on 3D-printed plastic inserts made from polyamide 12 (PA12) and glass bead-filled PA12 (PA12GB). The primary objective was to evaluate the impact of these surface treatments on the functional properties and service life of the mold inserts. A comprehensive analysis was conducted, including a detailed characterization of roughness using a confocal microscope, cross-sectional analysis to determine layer thickness, and tribological tests employing the ball-on-disc method to assess wear resistance. The study employed a modular injection mold and tested a range of surface finishing processes, including PostProcess Suspended Rotational Force (SRF) technology, metal decomposition coatings from HVM Plasma, and various methods from DyeMansion (Powershot S and Powerfuse). Results show a significant reduction in surface roughness across all methods. Notably, the vapor-based Powefuse treatment from DyeMansion achieved a surface roughness (R_a_) of 1.2797 μm, which is below the typical R_a_ value of 1.6 μm for conventional metal molds, thereby making it suitable for high-quality molding applications. The tribological analysis provided critical insights into the durability and wear resistance of the treated surfaces, supporting their potential for extended use. This research validates the potential of specific post-processing methods to transform AM parts into functional tooling, enabling cost-effective and rapid prototyping in the plastics industry.

## 1. Introduction

The advent of additive manufacturing (AM), commonly referred to as 3D printing, has revolutionized the fields of rapid prototyping and manufacturing. This technology enables the creation of complex geometries directly from a digital model, offering unparalleled design freedom and significantly reducing production lead times and costs compared to conventional subtractive methods [1,2,3]. One of the most promising, yet challenging, applications of AM is the production of functional tooling, specifically mold inserts for injection molding. While traditionally made from steel or aluminum, AM offers a viable alternative for producing low-volume prototypes and small-series parts [4,5].

Plastic injection molding is a cornerstone of modern manufacturing, a process that relies on a precisely designed mold to shape molten plastic into a final product [6]. The quality of the final part is inextricably linked to the quality of the mold’s surface. A high-quality surface finish ensures proper material flow, minimizes defects, and facilitates easy part ejection [7,8]. However, parts produced by most AM technologies, particularly those based on powder bed fusion, such as HP Multi Jet Fusion (Palo Alto, CA, USA), inherently possess a relatively high surface roughness due to the material’s layered deposition and sintered nature [9]. This roughness can directly transfer to the molded parts, leading to an unacceptable finish and potentially affecting dimensional accuracy [10,11]. Furthermore, the longevity of these plastic molds is limited by wear and tear caused by the high temperatures, pressures, and friction during the injection cycle.

In current industrial practice, polymer tooling and molds are commonly produced using additive manufacturing methods such as digital light processing (DLP), selective laser sintering (SLS), or emerging thermoset direct ink writing (DIW) techniques. These processes enable the fabrication of detailed geometries with relatively high resolution; however, they also present notable limitations. Parts produced by DLP and SLS often exhibit reduced geometric fidelity and higher surface roughness due to the effects of layer discretization and powder sintering. In contrast, DIW of thermoset materials is frequently constrained by anisotropic shrinkage, limited overhang capability, and post-curing distortions that compromise surface uniformity and dimensional accuracy [12,13]. These drawbacks underscore the need for further improvement in both process control and post-processing strategies, as addressed in the present study, which focuses on the surface treatment of MJF-produced polymer inserts for functional injection molding applications.

Various post-processing techniques have been developed to overcome these limitations and enhance the surface quality and durability of 3D-printed parts. These methods range from mechanical and chemical smoothing to the application of coatings. While some studies have explored these techniques, a comprehensive, comparative analysis of their effect on the functional properties of 3D-printed plastic molds for injection molding remains limited [14]. There is a specific need to evaluate the combined effects of these treatments on surface roughness and tribological performance, directly correlating these properties with the mold’s ability to produce quality parts over multiple cycles [15,16,17].

This paper aims to bridge this knowledge gap by investigating the surface treatment of 3D-printed plastic injection inserts. The primary objectives of this study are:

- To evaluate and compare the effectiveness of different surface smoothing technologies and coatings on the surface roughness of PA12 and PA12GB 3D-printed parts.
- To perform a detailed tribological analysis to quantify the wear resistance of the treated surfaces under conditions simulating the injection molding process.
- To analyze the thickness of the applied surface layers to understand their contribution to mold durability and design constraints.
- To correlate the treated molds’ physical properties (roughness, wear) with their functional performance in an injection molding machine, mainly focusing on plastic flowability and final part quality [18].

The research utilized a modular injection mold, enabling systematic and repeatable testing. The findings will provide valuable insights for engineers and manufacturers seeking to leverage additive manufacturing for cost-effective and efficient tooling solutions in the prototyping and small-series production of plastic components [19,20].

## 2. Materials and Methods

### 2.1. Methods-Injection Molding Process and Equipment

The core of this research is based on the plastic injection molding process, a thermodynamic cyclic forming method. The process involves converting plastic granules into a finished product under controlled heat and pressure. The sequence of operations is as follows:

1. Granule Feeding: Plastic granules are fed into the injection unit from a hopper.
2. Melting and Homogenization: A rotating screw transports the granules through a heated cylinder. The combination of heat from the cylinder and friction from the screw’s rotation melts and homogenizes the plastic into a viscous melt.
3. Injection: The screw moves axially, acting as a piston, to inject the molten plastic into the cavity of the closed mold.
4. Cooling and Solidification: The plastic cools and solidifies inside the mold.
5. Ejection: The mold opens, and ejector pins push the finished part out [21,22,23].

For this study, injection molding was performed on a fully electric, horizontally designed molding press. This machine is highly suitable for high-precision, automated production. Its all-electric nature ensures precise control over all movements, which is critical for consistent results in a research setting.

### 2.2. Materials-Mold Manufacturing via Additive Technology

**Figure 1:**
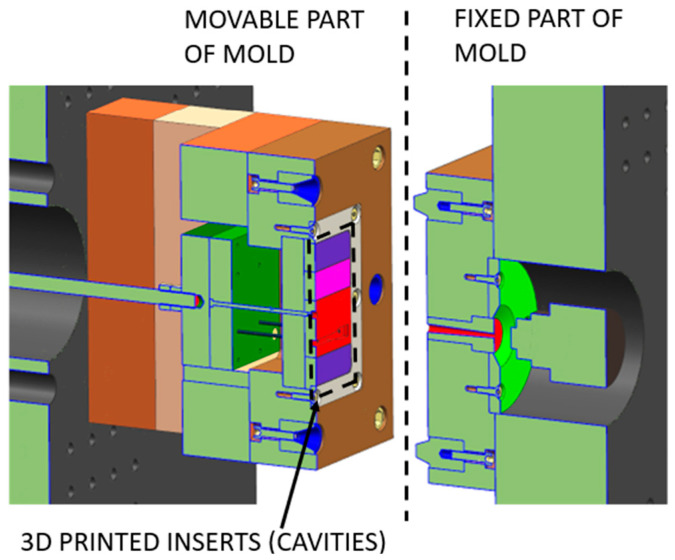
Schematic cutted view of the modular injection mold.

A “Modular Injection Mold” (Figure 1) was custom-designed to facilitate testing various inserts. The mold’s key feature is a reconfigurable frame accommodating different 3D-printed inserts. The design includes a movable and fixed part, each with a designated space for the inserts. This modularity enables the quick and efficient testing of various insert designs and surface treatments without requiring the manufacture of an entirely new mold for each test. The geometry of the insert is designed to be simple enough to minimize flow-related defects but complex enough to represent a real-world part [24,25].

The 3D-printed inserts were produced using HP’s Jet Fusion 3D 4200/5200 printers(USA, CA, Palo Alto). This technology, known as Multi Jet Fusion (MJF), is a powder bed fusion process that utilizes a proprietary fusing agent to bind powder particles [26] selectively. A thin layer of powder is first deposited and heated to a temperature just below the material’s melting point. A print head then jets fine droplets of a fusing agent onto the areas corresponding to the part’s geometry [27,28]. An infrared lamp then passes over the bed, causing the fusing agent to absorb heat and melt the underlying powder, solidifying the part layer by layer. This process is repeated until the part is complete. The parts were printed from PA12, a general-purpose semi-crystalline polyamide, and PA12GB, PA12 with a 40% glass bead filler. Adding glass beads increases the material’s stiffness and heat deflection temperature, making it potentially more suitable for high-stress applications, such as injection molding [29,30].

### 2.3. Characterization-Surface Treatment

This study investigated three main categories of surface treatment:

- Chemical Vapor Smoothing (DyeMansion)

The Powefuse process, provided by DyeMansion GmbH (Planegg, Germany), is a chemical vapor smoothing technique that utilizes a vaporized ecological liquid, primarily benzyl alcohol. The vapor condenses on the surface of the 3D-printed part, causing the top layer of the material to soften and reflow. This process effectively reduces surface roughness by filling voids and smoothing the layered texture [31]. The smoothing intensity can be controlled by adjusting the exposure time and vapor pressure. For this study, three intensity levels were tested: “Light”, “Balanced”, and “Strong”.

The Powershot C and Powershot S methods were also part of the finishing process. The Powershot C blasting system uses a granular medium to remove loose, unsintered powder from the part’s surface. The Powershot S is a smoothing system that uses a media blasting process with fine polymer beads to compact and smooth the surface without scratching it [32].

- Mechanical Smoothing by PostProcess (Buffalo, NY, USA)

The PostProcess RADOR Surface Finish system uses a patented Suspended Rotational Force (SRF) technology. This process combines optimized energy, vertical motion, and a specially formulated consumable to achieve the desired surface finish. The parts are subjected to a controlled tumbling process within a chamber, which mechanically smooths the surface.

- Metal Decomposition Coatings (HVM Plasma)

For a comparative durability analysis, samples were treated with thin metal decomposition coatings. This process involves the breakdown of a metal and its subsequent adhesion to the surface of the 3D-printed plastic. The following metals were applied:

Chromium (Cr): Known for its hardness and wear resistance.

Hafnium (Hf): Chosen for its high melting point and chemical stability.

Aluminum (Al): Selected for its lightweight and corrosion-resistant properties.

## 3. Results

### 3.1. Surface Roughness Measurement

Surface roughness was a key metric for evaluating the effectiveness of each treatment. Measurements were performed using an Olympus (Tokyo, Japan) LEXT confocal microscope. For each sample, the roughness was measured in two mutually perpendicular directions over a large area (10 fields of view by 10 fields of view) to ensure a statistically representative result. Each sample was scanned five times, and the data was averaged. The primary roughness parameter measured was the arithmetic mean roughness (R_a_) in accordance with the ISO 4287 standard.

**Figure 2:**
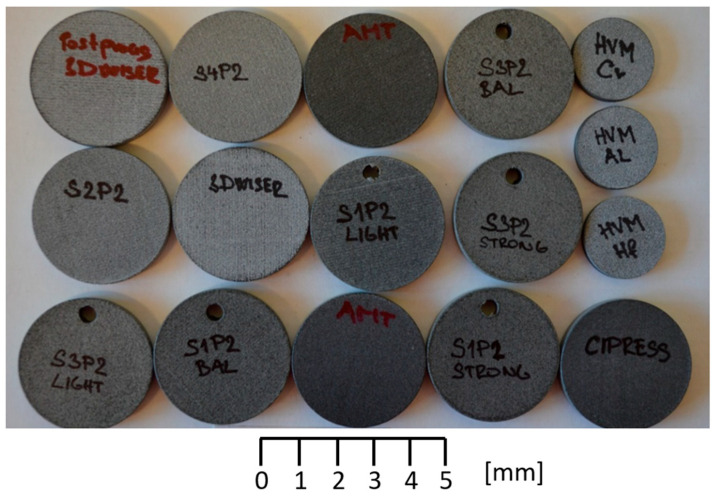
Surface quality testing specimens.

Considering the varying surface quality of the individual parts after smoothing, it was necessary to conduct a thorough analysis of the surface itself. All samples used for analysis are shown in Figure 2.

**Figure 3:**
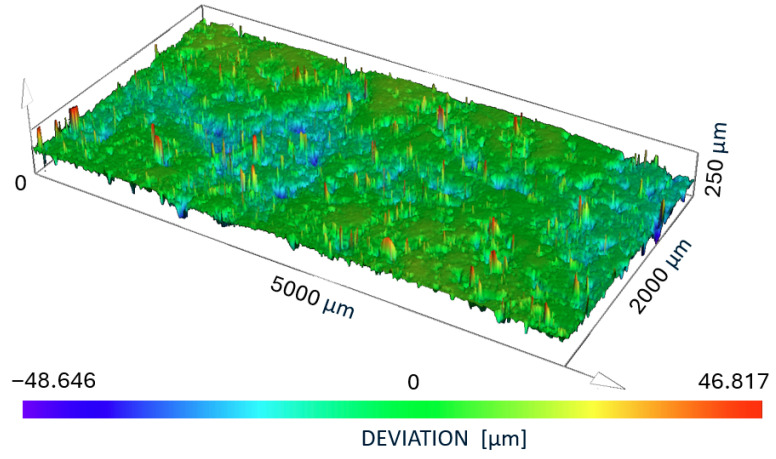
The results of the study on surface treatment using PostProcess.

An Olympus LEXT (Tokyo, Japan) confocal microscope was used to measure roughness. The measurement was performed in two mutually perpendicular directions, i.e., 10 fields of view × 10 fields of view. Each sample was scanned five times. The λc coefficient was automatically assigned to 800 μm. The analysis for sample alignment was performed using three points with noise peak reduction. The results of the study on surface treatment using PostProcess are presented in Figure 3.

### 3.2. Surface Layer Thickness

**Figure 4:**
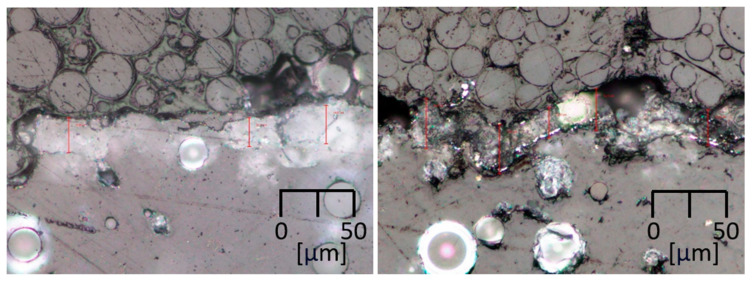
A microscopic image of a cross-section shows the interface between the base PA12 material and a surface-treated layer for the Postprocess technology (**left**) and HVM Chromium technology (**right**).

To assess the integrity and thickness of the applied coatings and smoothed layers, selected samples were cross-sectioned through their center. A metallographic grinding process was performed, with cold mounting to prevent any thermal influence on the sample. The thickness of the treated layer was then measured using microscopy. An example of this result is shown in Figure 4.

### 3.3. Tribological Testing

The wear resistance of the treated surfaces was quantified through a tribological analysis. The tests were conducted on an Anton Paar TRB3 (Graz, Austria) device using the “Ball on Disc” method. This setup simulates the frictional wear between the mold and the injected plastic. Key parameters were:

Rotational Speed: 600 rpm.

Standard Load: 20 N.

Friction Body: 6 mm diameter Alumina (Al_2_O_3_) balls.

Total Cycles: 160,000 cycles (equivalent to a total sliding distance of 5026.55m).

The weight loss of the samples was measured using a Sartorius Entris 224i-1S (Gottingen, Germany) scale, and the wear track width was analyzed to quantify the degree of wear.

### 3.4. Surface Roughness

The surface roughness measurements provided a clear quantitative comparison of the different treatment methods. The average R_a_ values for all samples are summarized in Table 1. All results are statistical average values for 10 samples of each type.

As shown in Table 2, the DyeMansion Powefuse method with the “Strong” setting (S3P2 STRONG) yielded the lowest surface roughness, with an average R_a_ value of 1.2797 μm. This is a significant improvement over the other methods. The standard for metal injection molds is typically R_a_ = 1.6 μm. Our best result is below this value, indicating a surface quality suitable for high-precision injection molding.

The differences in surface roughness values arise from the distinct mechanisms of each treatment—mechanical abrasion, vapor-induced surface reflow, or metal coating deposition—which affect the microstructure and surface topology differently. To address this, additional surface characterization, such as cross-sectional microscopy and tribological analysis, was performed to correlate roughness with coating thickness and wear behavior. These complementary methods provide a more comprehensive understanding of how each treatment modifies the surface and influences functional performance.

### 3.5. Surface Layer Thickness-Results

The thickness of the treated surface layer is a critical factor for both mold durability and the preservation of fine geometric details. The cross-sectional analysis yielded the following results, as summarized in Table 2.

The Powefuse method (S1P2 STRONG) yielded a very thin surface layer, indicating that the process primarily affects the outermost surface without significantly altering the overall dimensions. Conversely, the PostProcess and S2P2 methods resulted in thicker surface layers, which could impact the dimensional accuracy of the molded part.

### 3.6. Tribological Performance

The tribological tests provided data on the wear resistance of the treated surfaces. The primary outcome was the wear track width, which directly corresponds to the material removed during the test. The results are presented in Table 3.

The Powefuse (S3P2 STRONG) treated sample demonstrated the lowest wear track width and wear volume loss, indicating superior wear resistance among the plastic-based treatments. As expected, the HVM Plasma Cr coating also performed exceptionally well, showcasing the benefits of a hard metallic surface layer.

Metallic coatings have higher thermal conductivity and lower thermal expansion than PA12-based substrates, which can create minor local thermal stresses at the interface. However, given the thin coating layers (<30 µm) and moderate molding temperatures, these effects remain minimal and do not cause delamination.

The actual injection molding was performed to demonstrate its applicability. The main limitation was the surface degradation, which occurred after more than 200 cycles. This value is highly geometry-oriented, and it was tested for the cavity shown in Figure 2.

## 4. Discussion

The findings of this study highlight the crucial role of post-processing in realizing the full potential of 3D-printed plastic parts for injection molding applications. The untreated PA12 and PA12GB parts, due to their high intrinsic roughness, are unsuitable for producing cosmetic or functional parts where surface quality is a key consideration. The roughness values measured on these untreated parts (R_a_ typically above 6 μm) would lead to significant drag during part ejection and a poor finish on the final product.

The most compelling result is the performance of the DyeMansion Powefuse method. The “Strong” setting successfully reduced the surface roughness to a level (R_a_ = 1.2797 μm) suitable for injection molding and surpasses the typical standards for metal molds. This is a game-changing finding for the rapid prototyping industry, as it suggests that high-quality, professional-grade parts can be produced using cost-effective and quickly manufactured plastic molds. The mechanism behind this success is the precise control of the vapor-smoothing process, which effectively melts and reflows the outer layer of the material, filling in the microscopic valleys and reducing the peaks. The fact that this is achieved with minimal layer thickness addition (1.7 µm) is also significant, as it means the dimensional accuracy of the mold cavity is well-preserved.

In contrast, while providing improvements, the other tested methods did not reach the same level of surface quality. The mechanical smoothing from PostProcess and the other DyeMansion methods resulted in roughness values that are still too high for high-quality cosmetic parts. The metal decomposition coatings, particularly the aluminum coating, led to a higher roughness than some plastic-based treatments. This can be attributed to how the coating adheres to the already rough, porous surface of the 3D-printed part, which may not fill in the roughness but rather replicate and magnify it. Despite having a lower roughness, the chromium coating still fell short of the Powefuse method. This highlights that the initial surface preparation is paramount, while material hardness is essential for wear resistance.

The tribological results further support the superiority of the Powefuse treatment. The low wear volume loss and narrow wear track indicate that the treated surface is highly durable and resistant to the abrasive friction experienced during the injection molding process. This suggests that a mold treated with this method would have a longer service life than other treatments or untreated parts, allowing for more successful production cycles before replacement.

Future research will build upon these findings. Specifically, the following phases will focus on:

Extended Wear Testing: Long-term injection molding tests are conducted to determine the number of cycles a Powefuse-treated mold can withstand before degradation in part quality becomes noticeable.

Thermal Management: Investigating the effect of mold cooling on the final part’s dimensional accuracy. Plastic molds have lower thermal conductivity than metal molds, resulting in different cooling behavior. Optimizing cooling channels and cycles will be critical for high-volume production.

Alternative Materials and Processes: Continuing the research with other AM technologies, such as Voxeljet PMMA 3D printing and precision casting, to explore a broader range of materials and their suitability for tooling applications.

Flowability Analysis: A more in-depth analysis of plastic flowability inside the treated molds, correlating the measured surface roughness directly with melt flow behavior and part fill quality.

This research demonstrates that a proper surface treatment can elevate a fundamental 3D-printed part from a simple prototype to a functional, durable tool for manufacturing. The differences observed among the tested surface treatments can be attributed to the distinct ways in which each process modifies the polymer surface’s microstructure. Mechanical methods, such as the PostProcess SRF system, primarily act through controlled abrasion and compaction of surface asperities, resulting in partial smoothing but limited densification of the material. In contrast, the vapor-based Powefuse process induces localized surface melting and reflow, which fills interlayer voids and produces a denser, more homogeneous surface layer with significantly lower roughness. The plasma-deposited metallic coatings, on the other hand, create thin, hard films that enhance wear resistance but tend to replicate the underlying topography, thus preserving some of the initial roughness. These different mechanisms explain the variations in measured surface roughness and tribological performance, underscoring the importance of matching the treatment process to the desired balance between surface quality and durability.

The results of this study are consistent with recent findings on the durability of polymer molds produced by additive manufacturing. Several studies have shown that the service life of polymer AM molds is strongly influenced by surface integrity, thermal stability, and wear resistance, which can be significantly improved through targeted post-processing [26,27,32]. Similarly to our observations, these works reported that smoother and denser surface layers reduce frictional wear during molding cycles and delay thermal degradation. The present study extends this understanding by demonstrating that vapor-based surface treatment can achieve both low roughness and high wear resistance, thereby enhancing the practical durability of polymer molds for prototype and small-series injection molding applications.

## 5. Conclusions

This paper has summarized the potential of surface treatments for enhancing the functional properties of 3D-printed plastic molds used in prototype and small-series injection molding. We have demonstrated that not all post-processing methods are equally effective, as evidenced by roughness analysis, surface layer thickness measurement, and tribological testing.

The vapor-based Powefuse treatment from DyeMansion, specifically at its “Strong” intensity setting, proved to be the most effective method, achieving a surface roughness of R_a_ = 1.2797 μm. This value is below the standard for metal injection molds, validating the possibility of using 3D-printed plastic molds to produce high-quality parts. The tribological analysis further confirmed the superior wear resistance of this treatment, indicating a longer potential service life for the molds.

The findings confirm that, with appropriate post-processing, 3D-printed plastic molds can transition from a mere proof of concept to a practical, cost-effective solution for rapid tooling. This advancement will significantly benefit industries that require fast turnaround times for prototypes and low-volume production runs. Future research will build on these results by conducting real-world injection molding trials and exploring other AM technologies and materials to refine the process further and expand the capabilities of AM-based tooling. The research must continue with the testing of additional types of surface improvements and various AM production techniques.

## Figures and Tables

**Table 1:** Evaluation of Measured Roughness Values for all samples.

Sample	Roughness R_a_ [µm]	ISO 4287	Roughness R_y_ [µm]	Roughness R_z_ [µm]
PostProcess	4.0303	40.187	37.762	50.2628
Plasma—HVM AL	7.818	48.053	45.287	68.109
S1P2 STRONG	3.058	15.930	13.933	19.897
S1P2 LIGHT	3.168	17.936	15.667	21.056
S1P2 BAL	3.886	20.056	18.387	25.118
S2P2	4.066	26.418	21.771	29.871
S3P2 STRONG	1.2797	6.086	7.4969	9.5512
S3P2 LIGHT	3.1667	17.9358	15.667	21.056
S3P2 BAL	3.372	18.422	16.597	22.138
S4P2	4.541	27.001	23.291	31.875

**Table 2:** Measurement of Surface Layer Thickness.

Sample	Thickness [µm]
PostProcess	26.2
HVM_Cr	31.8
S1P2 STRONG	1.7
S2P2	40.8

**Table 3:** Tribological test results for selected samples.

Sample	Wear Track Width [µm]	Wear Volume Loss [mm^3^]
Untreated PA12	250	0.85
S1P2 LIGHT	210	0.65
S1P2 STRONG	185	0.58
HVM Plasma Cr	155	0.42
HVM Plasma Al	190	0.61
S3P2 STRONG	95	0.25

## Data Availability

The original contributions presented in this study are included in the article. Further inquiries can be directed to the corresponding author.

## Abbreviations

The following abbreviations are used in this manuscript:

MJF	Multi Jet Fusion
HP	Hewlett Packard
AM	Additive Manufacturing
PA	Polyamide
